# Pediatric Exclusivity Revenues for Cancer Drugs

**DOI:** 10.1001/jamapediatrics.2024.4449

**Published:** 2024-11-11

**Authors:** Ameet Sarpatwari, Liam Bendicksen, Douglas S. Hawkins, Lia Gore, Florence T. Bourgeois

**Affiliations:** 1 Program On Regulation, Therapeutics, And Law, Division of Pharmacoepidemiology and Pharmacoeconomics, Department of Medicine, Brigham and Women’s Hospital and Harvard Medical School, Boston, Massachusetts; 2 Department of Pediatrics, Seattle Children’s Hospital, University of Washington, Seattle, Washington; 3 Department of Pediatrics, University of Colorado School of Medicine and Center for Cancer and Blood Disorders, Children’s Hospital Colorado, Aurora; 4 Harvard–MIT Center for Regulatory Science, Harvard Medical School, Boston; 5 Department of Pediatrics, Harvard Medical School, Boston, Massachusetts; 6 Pediatric Therapeutics and Regulatory Science Initiative, Computation Health Informatics Program, Boston Children's Hospital, Boston, Massachusetts

## Abstract

This cohort study calculates the cost of pediatric trials and the value of pediatric exclusivity for select cancer drugs.

To spur pediatric drug research and product labeling, the US Congress established an incentive program under the Best Pharmaceuticals for Children Act, which provides 6 months of additional market exclusivity for brand-name drugs in return for manufacturers completing pediatric trials requested by the US Food and Drug Administration (FDA).^1^ From program inception in 2002 through 2023, 254 drugs received pediatric exclusivity, of which 42 (17%) were cancer treatments.^2^ Manufacturers of some of these cancer drugs contracted with the Children’s Oncology Group (COG), a National Cancer Institute–supported, nonprofit pediatric clinical trials network,^3^ to conduct the trials necessary to obtain exclusivity. To assess the value of these pediatric trial data, we estimated the cost of investment for pediatric trials and the revenue from the pediatric exclusivity for a sample of drugs.

## Methods

The study cohort comprised cancer drugs granted pediatric exclusivity between January 2010 (date of earliest available COG data) and June 2023, for which COG led at least 1 trial that contributed to exclusivity determination and for which revenue data were available for 6 months before generic entry. In accordance with the Common Rule, this cohort study was exempt from ethics review and informed consent requirement because it was not considered human participant research. We followed the STROBE reporting guideline.

For each drug, we identified all trials conducted to obtain pediatric exclusivity, including trials outside COG. Using information from COG on total payments made by manufacturers for execution of COG-led trials, we estimated costs to manufacturers for all FDA-requested trials (eMethods in Supplement 1). We used a 10% cost of capital to estimate the cost of investment.^4^

To estimate revenue from pediatric exclusivity, we first identified an actual or expected generic entry date for each drug after addition of the 6-month exclusivity period (eMethods in Supplement 1). Actual or estimated total revenues for this period were obtained from the SSR Health and Cortellis Drug Discovery Intelligence databases, respectively. Revenue from exclusivity was calculated assuming a 55% market share erosion rate,^5^ representing the percentage of brand-name-drug use replaced by generic-drug use during the first 6 months of competition.^4^

In sensitivity analyses, we used 5% and 15% cost-of-capital estimates and 40% and 70% market-share erosion rates. All costs and revenues were adjusted to 2022 US dollars. Data analysis was performed with Excel 16.89 (Microsoft).

## Results

Nine cancer drugs were granted pediatric exclusivity based on COG trials, of which 4 (44%) had the required revenue data for analysis: sunitinib, dasatinib, eribulin, and ruxolitinib. Two pediatric trials each were conducted for sunitinib and ruxolitinib, 3 for eribulin, and 5 for dasatinib (Table). Nine of these 12 trials (75%) were conducted by COG.

**Table.** Select Drugs Granted Pediatric Exclusivity Based on Children’s Oncology Group Trials

Generic (brand) name	Manufacturer	Original approval date	Pediatric exclusivity determination date	Actual or expected generic entry date	Total trials conducted for pediatric exclusivity	COG-led trials conducted for pediatric exclusivity	Estimated revenue from pediatric exclusivity, millions, $	Pediatric indication approved^a^
Sunitinib (Sutent)	Pfizer	January 26, 2006	February 7, 2019	August 2021	2	2	65	No
Dasatinib (Sprycel)	Bristol Myers Squibb	June 28, 2006	September 27, 2018	September 2024	5	3	389	Yes
Eribulin (Halaven)	Eisai	November 15, 2010	August 9, 2022	July 2027	3	2	42	No
Ruxolitinib (Jakafi)	Incyte	November 16, 2011	February 2, 2023	December 2028	2	2	741	No

Abbreviation: COG, Children’s Oncology Group.

^a^ Pediatric exclusivity is granted regardless of the safety and efficacy findings of the clinical trials.

Cost of investment to manufacturers for the pediatric trials was $156 million, corresponding to a mean (SD) of $13 ($11) million per trial and $39 ($12) million per exclusivity. Revenue from exclusivity totaled $1237 million, with a mean (SD) of $309 ($329) million per exclusivity and a range of $42 million for eribulin to $741 million for ruxolitinib (Figure). In sensitivity analyses, the total cost of investment ranged from $20 to $75 million per exclusivity and the revenue ranged from $228 million to $400 million per exclusivity.

**Figure.**
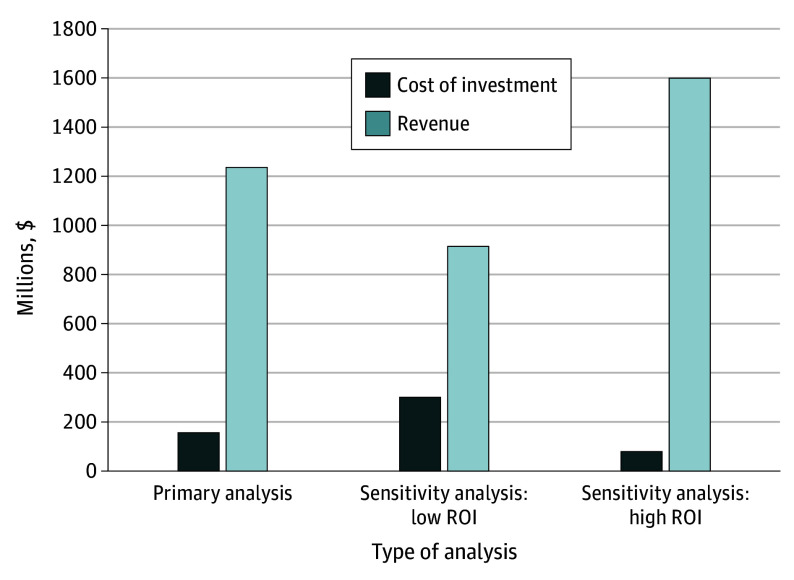
Per Exclusivity Cost of Investment for Pediatric Trials vs Pediatric Exclusivity Attributable Revenue The primary analysis assumed a 10% cost of capital and a 55% market-share erosion rate. The sensitivity analysis for lower return on investment (ROI) assumed a 5% cost of capital and a 70% market-share erosion rate. The sensitivity analysis for higher ROI assumed a 15% cost of capital and a 40% market-share erosion rate.

## Discussion

This cohort study of 4 cancer drugs granted pediatric exclusivity based on trials conducted with pediatric clinical trial networks found that exclusivity may generate substantial revenues. This finding highlights an opportunity to ensure that the value of pediatric exclusivity to manufacturers is commensurate with benefits to pediatric research programs. For example, manufacturers could invest a portion of returns in pediatric research enterprises, which have historically been underfunded.^6^

Study limitations included reliance on estimates for non-COG trial costs, projected revenue, and market-share erosion and focus on oncology drugs, which may limit generalizability of results to other drug types. Nonetheless, this study estimates the potential value of data generated by pediatric clinical trial networks, which could be leveraged to support further advances in pediatric care.

## Notes

Supplement 1.
eMethods.

Supplement 2.
Data Sharing Statement
