# Supplementary material for: Pediatric Exclusivity Revenues for Cancer Drugs
Source: JAMA Pediatr. 2024 Nov 11;179(1):91–3. doi: 10.1001/jamapediatrics.2024.4449 (PMC11555575; doi:10.1001/jamapediatrics.2024.4449)
Supplement: Supplement 1. — eMethods. [file jamapediatr-e244449-s001.pdf]

Supplementary Online Content

Sarpatwari A, Bendicksen L, Hawkins DS, Gore L, Bourgeois F. Pediatric exclusivity revenues for cancer drugs. *JAMA Pediatr*. Published online November 11, 2024. doi:10.1001/jamapediatrics.2024.4449

eMethods.

This supplementary material has been provided by the authors to give readers additional information about their work.

© 2024 Sarpatwari A et al. *JAMA Pediatrics.*

eMethods.

Manufacturer Costs for Trials Conducted to Obtain Pediatric Exclusivity

To estimate drug manufacturers' costs for the clinical trials conducted to obtain pediatric exclusivity, we used contract data provided by the Children's Oncology Group (COG). This information included the total payments made by manufacturers to COG to conduct the FDA-requested trials. We treated the total payment amount as reflecting 2012 dollars, the median start year of the COG-led trials, and adjusted it to 2022 US dollars using the consumer price index for all urban consumers.

<u>COG-led trials</u>

The cost of investment for COG-led trials was calculated as the total manufacturer cost of the trials x (1+the cost of capital)^(the number of years between the median start of the trials and the median end of pediatric exclusivity). The cost of capital accounts for manufacturers' opportunity costs—the value of manufacturers' forgone investments—and was set to 10% in the primary analysis, consistent with prior studies. In sensitivity analyses, we used 5% and 15% cost of capital estimates.

We estimated the standard deviation of the cost of investment for all trials as the standard deviation of the cost of investment for COG-led trials.

<u>Non-COG-led trials</u>

We estimated the cost of non-COG-led trials by first calculating the per-person, manufacturer cost of COG-led trials For each non-COG-led trial, we determined the total number of participants enrolled using information in FDA documents and ClinicalTrials.gov, and multiplied the per-person trial cost by the number of trial participants. The cost of investment for each non-COG-led trial was then calculated as the total cost of the trial x (1+the cost of capital)^(the number of years between the start of the trials and the end of pediatric exclusivity). As with COG-led trials, we used a 10% cost of capital for the primary analyses and 5% and 10% cost of capital estimates in sensitivity analyses.

© 2024 Sarpatwari A et al. *JAMA Pediatrics.*

Estimating Manufacturer Revenue from Pediatric Exclusivity

Expected Generic Entry Date

We estimated the market entry of generic drugs using settlement dates or, in their absence, the last expiring drug substance or product patent granted pediatric exclusivity.

Inflation Adjustment of Estimated Future Revenues

We adjusted estimated future revenues using the following formula: 2022 revenue=future revenue/(1+inflation rate)^number of years. We used SmartAsset's analysis of historical trends to project the inflation rate.

© 2024 Sarpatwari A et al. *JAMA Pediatrics.*
