# Supplementary material for: Pediatric Exclusivity Revenues for Cancer Drugs
Source: JAMA Pediatr. 2024 Nov 11;179(1):91–3. doi: 10.1001/jamapediatrics.2024.4449 (PMC11555575; doi:10.1001/jamapediatrics.2024.4449)
Supplement: Supplement 2. — Data Sharing Statement [file jamapediatr-e244449-s002.pdf]

Data Sharing Statement

Sarpatwari. Pediatric Exclusivity Revenues for Cancer Drugs. *JAMA Pediatr*. Published November 11, 2024. doi:10.1001/jamapediatrics.2024.4449

Data
Data available: Yes
Data types: Data (not involving human participants)
How to access data: Data will be made available upon request to the corresponding author (asarpatwari@bwh.harvard.edu)
When available: With publication

Supporting Documents
Document types: None

Additional Information
Who can access the data: Researchers whose proposed use of the data has been approved
Types of analyses: For any purpose
Mechanisms of data availability: With investigator support
